# Breast and Lung Cancer Screening Among Medicare Enrollees During the COVID-19 Pandemic

**DOI:** 10.1001/jamanetworkopen.2022.55589

**Published:** 2023-02-03

**Authors:** Christopher Doan, Shuang Li, James S. Goodwin

**Affiliations:** 1 Department of Medicine, University of Texas Medical Branch, Galveston; 2 Sealy Center on Aging, University of Texas Medical Branch, Galveston

## Abstract

**Importance:**

Several studies reported sharp decreases in screening mammography for breast cancer and low-dose computed tomographic screening for lung cancer in the early months of the COVID-19 pandemic, followed by a return to normal or near-normal levels in the summer of 2020.

**Objective:**

To determine the observed vs expected mammography and low-dose computed tomographic scan rates from the beginning of the pandemic through April 2022.

**Design, Setting, and Participants:**

In this retrospective cohort study assessing mammography and low-dose computed tomography rates from January 2017 through April 2022, data for January 2016 to February 2020 were used to generate expected rates for the period March 2020 to April 2022. The study included a 20% national sample of Medicare fee-for-service enrollees among women aged 50 to 74 years for mammography, and men and women aged 55 to 79 years for low-dose computed tomographic scan.

**Main Outcomes and Measures:**

Receipt of screening mammography or low-dose computed tomographic scan.

**Results:**

The yearly cohorts for the mammography rates included more than 1 600 000 women aged 50 to 74 years, and the cohorts for the low-dose computed tomographic scan rates included more than 3 700 000 men and women aged 55 to 79 years. From January 2017 through February 2020, monthly mammography rates were flat, whereas there was a monotonic increase in low-dose computed tomographic scan rates, from approximately 500 per million per month in early 2017 to 1100 per million per month by January 2020. Over the period from March 2020 to April 2022, there were episodic drops in both mammography and low-dose computed tomographic scan rates, coincident with increases in national COVID-19 infection rates. For the periods from March 2020 to February 2020 and March 2021 to February 2022, the observed low-dose computed tomographic scan rates were 24% (95% CI, 23%-24%) and 14% (95% CI, 13%-15%) below expected rates, whereas mammography rates were 17% (95% CI, 17%-18%) and 4% (95% CI, 4%-3%) below expected.

**Conclusions and Relevance:**

In this cohort study, the decreases in cancer screening during the early phases of the COVID-19 pandemic did not resolve after the initial pandemic surges. Successful interventions to improve screening rates should address pandemic-specific reasons for low screening participation.

## Key Points

**Question:**

Did screening for breast and lung cancer return to expected rates after the initial phase of the COVID-19 pandemic in 2020?

**Findings:**

In this cohort study of Medicare enrollees from the beginning of the COVID-19 pandemic in March 2020 through July 2022, both mammography and low-dose computed tomographic screening fell below expected rates, coincident with increases in national COVID-19 infection rates.

**Meaning:**

Interference with cancer screening by periodic surges in COVID-19 infections is a continuing problem.

## Teaser

This cohort study examines the observed vs expected mammography and low-dose computed tomographic scan rates from the beginning of the COVID-19 pandemic through April 2022.

## Introduction

Early in the COVID-19 pandemic, rates for all cancer screening tests were severely depressed, including mammography, colonoscopy and low-dose computed tomography (LDCT).^1,2,3,4,5,6,7,8,9^ The decrease in screening resulted in decreases in the rates of cancer diagnoses; specifically, a lower rate of diagnoses of early-stage cancer.^3,10,11^ This early decrease in screening was followed by a period of recovery, with rates returning to normal or near-normal in the summer of 2020.^1,4,6,12,13,14,15^

There are several limitations to the current information on cancer screening during the pandemic. First, screening rates have not been well studied beyond early 2020. One study using national claims data from March 2020 to February 2021 found a return to near-normal mammography rates during July through October 2020, but then another decline in January and February 2021.^4^ Second, many existing reports are from single institutions or from hospital systems, and have produced somewhat conflicting results. Third, the studies on cancer screening rates have not considered the temporal trends in use in the years before the pandemic. Whereas mammography rates were mostly flat,^6^ LDCT rates steadily increased over that time.^16,17,18,19^

Our objective was to use national Medicare data to assess rates of mammography and LDCT screening from January 2017 through July 2022, to determine if screening rates remained depressed later in the pandemic. We graphed unadjusted monthly rates from January 2017 through July 2022. We also calculated expected rates for March 2020 to February 2021 and March 2021 to February 2022, based on rates from January 2017 to February 2020, and compared those with the actual rates of screening.

## Methods

### Data Source

We used enrollment and claims data for a 20% national sample of Medicare beneficiaries between March 2016 and April 2022, with data updates through October 14, 2022. This included Medicare Beneficiary Summary files, Inpatient Claims files, Skilled Nursing Facilities Claims files, Outpatient Claims files, and Medicare Carrier files. The study protocol was reviewed and approved by the University of Texas Medical Branch institutional review board, which waived the need for written informed consent. It followed Strengthening the Reporting of Observational Studies in Epidemiology (STROBE) reporting guidelines for observational cohort studies (eFigures 1 and 2 in Supplement 1). All data were obtained via a data use agreement with the Center for Medicare and Medicaid Services, which prohibits data dissemination to others.

### Cohorts

For the descriptive analyses, we developed 64 monthly cohorts of beneficiaries who were alive on the first day of each month from January 2017 to April 2022. The mammography cohort was comprised of female enrollees aged 50 to 74 years who were alive on the first day of the month studied, and the LDCT cohort was comprised of male and female enrollees aged 55 to 79 years on the first day of the month studied. For the regression analyses, we constructed 5 12-month cohorts, starting in March of each year from 2017 to 2021 and ending the following February. Both the descriptive and the analytic cohorts were restricted to those with compete Medicare Parts A and B and no health maintenance organization (HMO) enrollment during 1 year prior and 1 year after the period studied. If the beneficiary died within the year after March 1, we kept the beneficiaries who had complete insurance until the death date. For each cohort, Agency for Healthcare Research and Quality (AHRQ) comorbidity scores were obtained from the previous year’s claims.^20^

### Beneficiary Characteristics

We used Medicare enrollment files to provide information on beneficiary age, sex, race and ethnicity, and Medicaid enrollment. The percentage of high school graduates in the patients’ zip code was obtained from the 2019 American Community Survey estimates of the US Census Bureau.^21^ We defined a beneficiary as having an identifiable primary care physician if the patient saw the same generalist physician (family medicine, internal medicine, general practice, or geriatrics) on 3 or more occasions in an outpatient evaluation and management setting (Current Procedural Terminology [CPT] codes 99201-99205 and 99211-99215) using the previous year’s claims.^22^ We used the AHRQ’s *International Statistical Classification of Diseases, Tenth Revision, Clinical Modification (ICD-10-CM)* algorithm to create a summary score for 38 comorbidities in the Elixhauser comorbidity indicator, using the previous year claims.^20^ We categorized the number of comorbidities into 3 groups (<0, 0, and >0).

### Outcomes

We identified screening mammography as a bilateral mammogram with CPT codes 77056, 77057, 77066, 77067 or 2022 Healthcare Common Procedure Coding System (HCPCS) codes G0202 or G0204. We then excluded beneficiaries with any mammogram (CPT codes 77055, 77056, 77057, 77065, 77066, 77077 or HCPCS codes G0202, G0204, G0206) in the 11 months before the index mammography. For lung cancer screening, outcomes were receipt of LDCT (CPT codes G0297, S8032, 71271) in enrollees who had not had a chest CT in the prior 11 months.^23^ Chest CTs were identified by CPT/ HCPCS codes (G0297, S8032, 71271, 71250, 71260, 71270) or ICD-10-CM codes BW2400Z, BW240ZZ, BW2410Z, BW241ZZ, BW24Y0Z, BWZ4YZZ, BW24ZZZ, BW2500Z, BW250ZZ, BW2510Z, BW251ZZ, BW25Y0Z, BW25YZZ, or BW25ZZZ.

### Statistical Analysis

We calculated the proportion of relevant Medicare beneficiaries who underwent mammography or LDCT in each month from January 2017 through April 2022. The denominator of the proportion was the number of relevant enrollees who were alive on the first day of each month and the numerator was the number of those enrollees undergoing the screening test during that month. We tested for a change in slope for monthly mammography and LDCT rates from January 2017 to February 2020 using the statistical software Joinpoint (version 4.9.1.0, National Cancer Institute).^24,25^ We downloaded the US daily COVID-19 case data from the Our World in Data website, then calculated the cumulative rate per 1 million for each month from January 2020 to July 2022.^26^ We also described the beneficiary characteristics for the yearly cohorts. To investigate the change in screening rates during the COVID-19 pandemic, we built prediction models using the 2017 to 2019 yearly cohorts to predict the rate of mammography and LDCT for the 2020 and 2021 cohorts. We built a linear probability model for each subcohort, with year in the model. We then predicted the expected rate of mammography and LDCT with 95% CIs, and calculated the observed/expected ratio (O/E ratio) for the periods March 1, 2020, to February 28, 2021, and March 1, 2021, to February 28, 2022. In another analysis, we used a logistic regression model with 2017 to 2019 data to generate the expected rates and 95% CIs with the bootstrap method of mammography and LDCT for March 1, 2020, to February 28, 2021. In this model, we included all enrollee characteristics described herein. We limited the logistic regression to predicting the 2020 expected rates, because the look-back period for the 2021 cohort was (March 1, 2020 to February 28, 2021) occurred during the first year of the pandemic, when medical care was disrupted and the enrollee characteristics such as comorbidities based on claims during that period would be affected. All analyses were performed using SAS Enterprise statistical software (version 7.1, SAS Institute) at the Centers for Medicare & Medicaid Services Virtual Research Data Center.

## Results

The characteristics of the mammography and LDCT cohorts are presented in eTables 1 and 2 in Supplement 1. Separate cohorts were created for 5 12-month periods, from March through the following February, starting in March 2017 and ending in February 2022. For mammography, the yearly cohorts were more than 1 600 000 women aged 50 to 74 years, with 7.6% to 9.6% Black, 5.3% to 5.6% Hispanic, 79.4% to 80.5% white, and 5.4% to 6.6% other race/ethnicity (including Asian/Pacific Islanders, American Indian/Alaska native, or others). The yearly LDCT cohorts were more than 3 700 000 men and women aged 55 to 79 years, with 45.5% to 45.9% male, 6.8% to 8.3% Black, 4.9% to 5.2% Hispanic, 80.7% to 81.5% white, and 5.6% to 6.8% other race/ethnicity (including Asian/Pacific Islanders, American Indian/Alaska native or others). The number of enrollees in the yearly cohorts declined over time (for example, from 3 875 754 in 2018 to 3 719 971 in the LDCT cohorts, a 4% decrease). This was due to an increasing enrollment of Medicare enrollees in HMOs, which were excluded from the cohorts (eTable 3 in Supplement 1).

**Figure 1.**
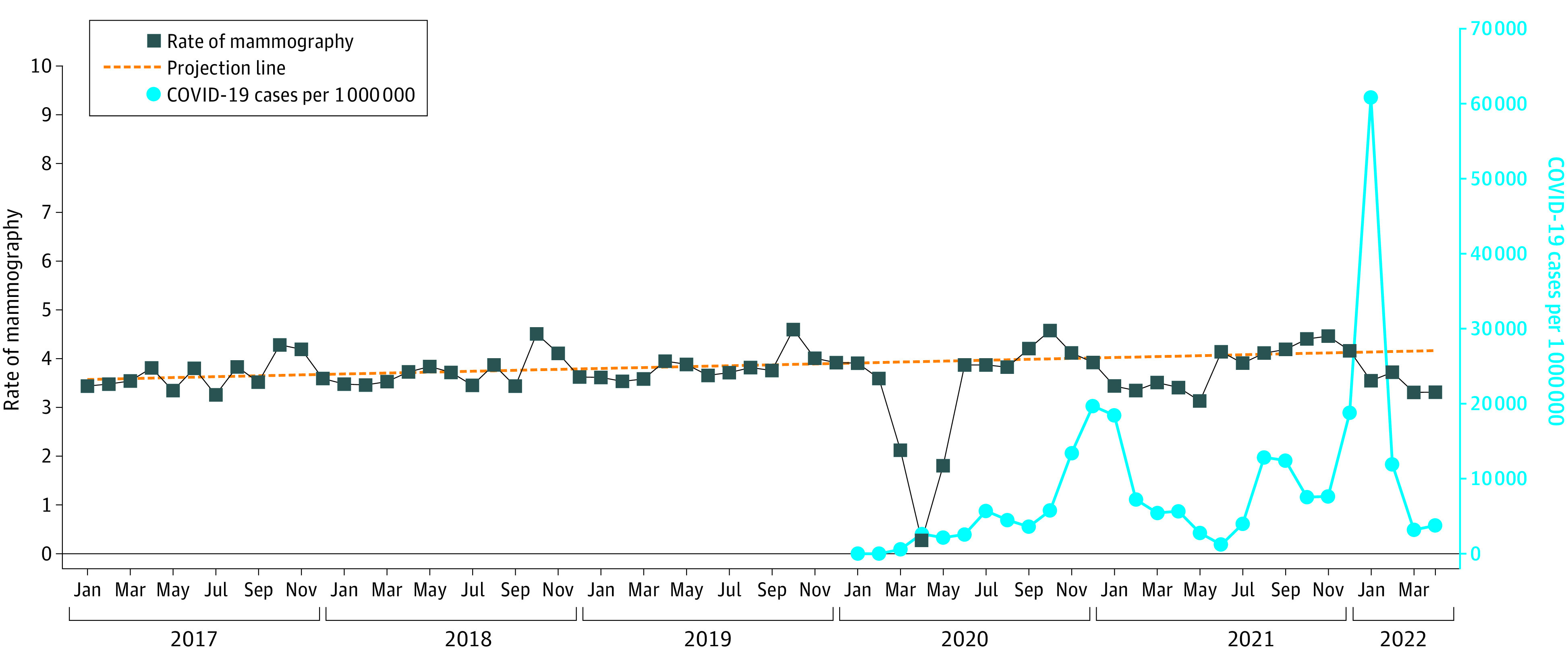
Rates of Monthly Mammography Screening From January 2017 Through July 2022 for Women Aged 50 to 74 Years With Fee-for-Service Medicare Monthly national COVID-19 infection rates from January 2020 through July 2022 are also shown. The monthly rates were adjusted to reflect 30 days per month. The dotted prediction line was generated from the 38 months from January 2019 through February 2020. The slope from January 2017 to February 2020 was 0.00838% (95% CI, −0.0015% to 0.01830%) per month, not significantly different from 0. There were no significant changes in slope over that same period by joinpoint analyses. There were increases in rates each October, corresponding to Breast Cancer Awareness Month. After the initial fall in rates in March, April, and May of 2020, there were periodic decreases in screening rates, corresponding to increases in national COVID-19 case rates.

**Figure 2.**
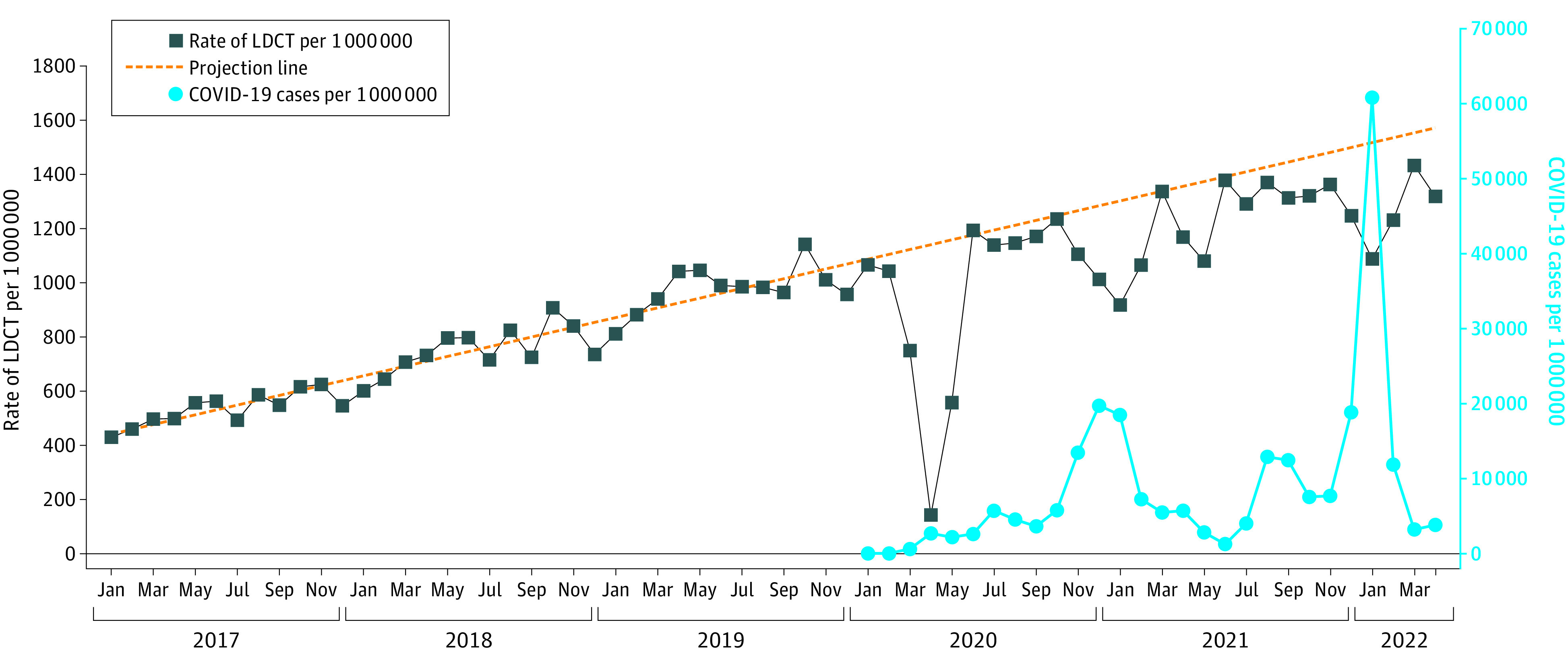
Monthly Rates of Low-Dose Computed Tomographic (LDCT) Lung Cancer Screening Among Medicare Fee-for-Service Beneficiaries Aged 55 to 79 Years, From January 2017 to July 2022 We adjusted the monthly LDCT rates to 30 days/mo. The dotted prediction line was generated from the 38 months from January 2017 through February 2020. There were no significant changes in slope over that same period by joinpoint analyses. National COVID-19 infection rates are given starting in 2020. The slope of the regression line was an increase in 17.81 (95% CI 15.65-19.96) LDCTs per million enrollees per month. As with the mammography rates, there were periodic decreases in LDCT screening rates corresponding to increases in national COVID-19 care rates.

Figure 1 and Figure 2 graph the unadjusted monthly rates of mammography and LDCT lung cancer screenings from January 2017 through July 2022. Also shown in the figures is the monthly national rate of COVID-19 infections. During 2017, 2018, and 2019, mammography rates were flat, whereas there was a steady increase in LDCT rates, from approximately 500 per million enrollees per month in early 2017 to approximately 1100 per million in January 2020. The figures also show the regression lines produced by the mammography and LDCT rates for the months from January 2017 through February 2020, then those projected over the months from March 2020 to July 2022. A Jointpoint analysis revealed no change in the slopes over the 38 prepandemic months from January 2017 through February 2020. Starting in March 2020, there were profound decreases in the rates of both screening tests, as previously reported. These rates recovered by June 2020, but later in 2020 and in 2021 and early 2022, the monthly rates of both screening tests were below the projected rates. For example, in January 2021, the LDCT rate was 30.5% lower than the projected rate, and in January 2022, it was 28.3% lower. Both decreases were coincident with increases in national COVID-19 infection rates. Mammography rates had a similar pattern, but with smaller dips in rates. For example, in January 2021 the mammography rate was 14.6% lower than projected, and in January 2022, it was 14.2% lower.

We calculated the observed and expected rates for mammography and LDCT for March 2020 to February 2021 and for March 2021 to February 2022, based on linear probability models generated from the 2017, 2018, and 2019 cohorts. As shown in Table 1, the observed mammography rate was 17% lower than expected (O/E ratio, 0.83; 95% CI, 0.82-0.83) for the period March 2020 to February 2021. For March 2021 to February 2022, the rate for mammography was 4% lower (O/E ratio, 0.96; 95% CI, 0.96-0.97).

**Table 1.** Expected and Observed Mammogram Rates for March 20 to February 21 and March 21 to February 22 From a Prediction Model Using the 2017 to 2019 Cohorts^a^

Characteristic	March 20 to February 21			March 21 to February 22		
	Mammogram rate		O/E ratio (95% CI)	Mammogram rate		O/E ratio (95% CI)
	Expected, % (95% CI)	Observed, %		Expected, % (95% CI)	Observed, %	
All	47.79 (47.68-47.91)	39.53	0.83 (0.82-0.83)	48.92 (48.75-49.08)	47.11	0.96 (0.96-0.97)
Age, y						
50-65	35.15 (34.88-35.40)	27.50	0.78 (0.78-0.79)	35.56 (35.19-35.93	32.98	0.93 (0.92-0.94)
66-70	50.70 (50.54-50.87)	42.31	0.83 (0.83-0.84)	51.69 (51.45-51.92)	49.83	0.96 (0.96-0.97)
71-74	49.51 (49.31-49.71)	40.91	0.83 (0.82-0.83)	50.78 (50.49-51.07)	48.80	0.96 (0.96-0.97)
Race						
Black	44.57 (44.19-44.95)	37.15	0.83 (0.83-0.84)	45.48 (44.93-46.02)	43.80	0.96 (0.95-0.97)
Hispanic	38.28 (37.80-38.75)	29.29	0.77 (0.76-0.77)	38.90 (38.22-39.58)	36.22	0.93 (0.92-0.95)
White	48.98 (48.85-49.11)	40.83	0.83 (0.83-0.84)	50.15 (49.97-50.34)	48.33	0.96 (0.96-0.97)
Other^b^	45.36 (44.89-45.82)	34.96	0.77 (0.76-0.78)	46.55 (45.87-47.22)	44.77	0.96 (0.95-0.98)
Medicaid						
No	50.80 (50.68-50.93)	42.45	0.84 (0.83-0.84)	51.90 (51.72-52.08)	50.08	0.96 (0.96-0.97)
Yes	31.61 (31.35-31.87)	23.26	0.74 (0.73-0.74)	31.80 (31.43-32.17)	28.74	0.90 (0.89-0.91)
PCP						
No	45.82 (45.68-45.96)	37.72	0.82 (0.82-0.83)	46.99 (46.79-47.19)	45.78	0.97 (0.97-0.98)
Yes	52.57 (52.37-52.78)	43.94	0.84 (0.83-0.84)	53.92 (53.63-54.22)	51.59	0.96 (0.95-0.96)
Comorbidity score						
<0	51.58 (51.38-51.77)	42.60	0.83 (0.82-0.83)	53.00 (52.72-53.28)	50.71	0.96 (0.95-0.96)
0	46.62 (46.45-46.79)	38.26	0.82 (0.82-0.83)	47.52 (47.27- 47.77)	46.04	0.97 (0.96-0.97)
>0	43.68 (43.43-43.92)	36.89	0.84 (0.84-0.85)	44.63 (44.27-44.98)	43.72	0.98 (0.97-0.99)

Abbreviations: O/E, observed/expected; PCP, primary care physician.

^a^ A linear probability model was used for each subcohort, with year as the only variable in the models.

^b^ Other includes Asian/Pacific Islanders, American Indian/Alaska native or others.

Table 1 also shows the observed vs expected rates of mammography in 2020 and 2021 stratified by enrollee characteristics. Each characteristic was analyzed in a separate model, with year as the only variable. Medicare enrollees aged 50 to 65 years, who comprised those on disability or with end-stage renal disease, had lower than expected observed rates (eg, O/E ratio, 0.78; 95% CI, 0.78-0.79) during March 2020 to February 2021, vs O/E ratio of 0.83 (95% CI, 0.83-0.84) for enrollees aged 66 to 70 years. Hispanic women had lower recovery rates; for example, in March 2020 to February 2021, O/E ratio, 0.77 (95% CI, 0.76-0.77) for Hispanics vs 0.83 (95% CI, 0.83-0.84) for White participants. Medicaid-eligible enrollees also had lower recovery rates. It is also notable that the expected rates for March 2020 to February 2021 and March 2021 to February 2022 were substantially lower for younger and Hispanic women and for those eligible for Medicaid, because their rates in the years prior to the pandemic were lower.

Table 2 presents similar analyses for LDCT. The observed rate for LDCT during March 2020 to February 2021 was 24% (95% CI, 23%-24%) lower than expected and, during March 2021 to February 2022, it was 14% (95% CI, 13%-15%) lower than expected. The results of the analyses stratified by enrollee characteristics were also similar to those for receipt of mammography. eTables 3 and 4 in Supplement 1 present stratified analyses for observed vs expected rates for mammography and LDCT, respectively, generated from a logistic regression model controlling for all variables in Table 1. We present only analyses for the March 2020 to February 2021 period because the lookback period for March 2021 to February 2022 was used to generate some of the variables included the first year of the pandemic, when medical care was disrupted, and the numbers of encounters and diagnoses in Medicare claims might have different implications. In the multivariable analyses, the patterns of the results were similar to the stratified analyses shown in Table 1 and Table 2, with younger age, Hispanic ethnicity, and Medicaid eligibility independently associated with lower observed vs expected rates during March 2020 to February 2021.

**Table 2.** Expected and Observed LDCT Rates for March 20 to February 21 and March 21 to February 22 From a Prediction Model Using the 2017 to 2019 Cohorts^a^

Characteristic	March 20 to February 21			March 21 to February 22		
	LDCT rate per 1 000 000		O/E ratio (95% CI)	LDCT rate		O/E ratio (95% CI)
	Expected (95% CI)	Observed		Expected per 1 000 000 (95% CI)	Observed	
All	14 939 (14 782-15 096)	11 411	0.76 (0.76-0.77)	17 666 (17 445-17 887)	15 201	0.86 (0.85-0.87)
Age, y						
55-65	26 376 (25 780-26 972)	19 690	0.75 (0.73-0.76)	31 339 (30 502-32 176)	25 699	0.82 (0.80-0.84)
66-70	17 093 (16 823-17 364)	13 383	0.78 (0.77-0.80)	20 198 (19 818-20 578)	18 012	0.89 (0.87-0.91)
71-75	14 069 (13 798-14 339)	10 670	0.76 (0.74-0.77)	16 712 (16 330-17 094)	14 293	0.85 (0.84-0.87)
76-79	4690 (4486-4894)	3944	0.84 (0.81-0.88)	5612 (5325-5898)	5943	1.06 (1.01-1.11)
Sex						
Male	16 544 (16 301-16 787)	12 790	0.77 (0.76-0.78)	19 577 (19 234-19 920)	16 913	0.86 (0.85-0.88)
Female	13 587 (13 383-13 791)	10 253	0.75 (0.74-0.77)	16 057 (15 769-16 346)	13 772	0.86 (0.84-0.87)
Race						
Black	10 714 (10 239-11 191)	7983	0.75 (0.71-0.78)	12 610 (11 938-13 282)	11 289	0.89 (0.85-0.94)
Hispanic	5892 (5447-6337)	4174	0.71 (0.66-0.77)	6908 (6280-7535)	6389	0.92 (0.85-1.02)
White	16 350 (16 167-16 532)	12 513	0.77 (0.76-0.77)	19 340 (19 083-19 560)	16 492	0.85 (0.84-0.86)
Other^b^	9409 (8907-9911)	7178	0.76 (0.72-0.81)	11 189 (10 479-11 899)	10 019	0.89 (0.84-0.95)
Medicaid						
No	14 008 (13 845-14 171)	10 886	0.78 (0.77-0.79)	16 567 (16 336-16 798)	14 546	0.88 (0.86-0.89)
Yes	21 173 (20 668-21 679)	15 154	0.72 (0.70-0.73)	25 144 (24 435-25 854)	20 288	0.81 (0.78-0.83)
PCP						
No	13 813 (13 633-13 993)	10 480	0.76 (0.75-0.77)	16 398 (16 143-16 653)	14 470	0.88 (0.87-0.90)
Yes	17 586 (17 278-17 894)	13 632	0.77 (0.76-0.79)	20 725 (20 292-21 159)	17 649	0.85 (0.83-0.87)
Comorbidity score						
<0	17 160 (16 846-17 474)	13 133	0.77 (0.75-0.78)	20 279 (19 836-20 722)	17 725	0.87 (0.85-0.89)
0	10 017 (9826-10 207)	7234	0.72 (0.71-0.74)	11 797 (11.525- 12.070)	10 577	0.90 (0.88-0.92)
>0	20 636 (20 277-20 995)	16 357	0.79 (0.78-0.81)	24 400 (23 895-24 906)	21 232	0.87 (0.85-0.89)

Abbreviations: LDCT, low-dose computed tomography; O/E, observed/expected; PCP, primary care physician.

^a^ A linear probability model was used for each sub-cohort, with year as the only variable in the models.

^b^ Other includes Asian/Pacific Islanders, American Indian/Alaska native, or others.

In sensitivity analyses, we repeated the analyses in Table 1 and Table 2, while stratifying the sample by age younger than 65 vs those aged 65 years or older (eTables 6-9 in Supplement 1) and by Medicaid eligibility (eTables 10-13 in Supplement 1). The pattern of results for mammography and LDCT rates in the different strata did not vary substantially from what was found in the entire cohort, but enrollees younger than 65 years had somewhat greater depression of screening rates in 2021 and 2022 than did those aged 65 years or older.

## Discussion

Almost all of the published reports on the effect of the COVID-19 pandemic on cancer screening focus on 2020, with most studies limited to the first 6 or 9 months of that year.^3,5,6,8,9,11,13,14^ The message from those studies was that there was a profound decrease in cancer screening rates early in the pandemic that was mostly or totally reversed by the summer of 2020. The major finding of the present study is a continued decrease in screening rates in 2021 and into early 2022. The lower-than-expected mammography and LDCT rates in 2020 and 2021 were not uniform throughout those years. Rather, there were months with rates at or very close to the predicted values, interspersed with months with values clearly lower than expected. The periods of lower-than-expected LDCT rates were coincident with surges in national COVID-19 infection rates.

Mafi et al^4^ studied the use of 6 ambulatory care services, including mammography, in US adults enrolled in commercial and public insurance plans from January 2019 to February 2021. They found a return to near-normal rates in the Medicare population during the summer and fall of 2020, followed by a dip in rates in January and February of 2021. They also noted that Medicaid eligibility was associated with lower rates of recovery in ambulatory services after the initial dip in the spring of 2020.^4^ Our findings show that the association of lower screening rates with surges in COVID-19 persisted into late 2021 and early 2022.

In the 38 months prior to the pandemic, mammography rates were flat; the slope of the monthly rates was not different from 0. In contrast the rates of LDCT screening rose in a linear fashion between January 2017 and January 2020, for an overall 148% increase. Accordingly, for the analyses of screening rates during the pandemic, we first estimated the expected values for screening assuming the rates would continue the patterns seen in the 38 prepandemic months, and compared those expected rates to the observed actual rates. After the initial depression of rates in early 2020, LDCT rates did approach the expected rates in months with low COVID-19 rates, but were depressed in months with higher COVID-19 rates.

Our findings that low income, as evidenced by Medicaid eligibility, and historically marginalized race/ethnicity were associated both with decreased prepandemic screening rates and impaired recovery of rates after the decline in March and April 2020 are consistent with prior reports.^4,8,9^ Additional resources for specific initiatives targeting those populations are necessary.^27^

The US Preventive Services Task Force broadened the indications for LDCT screening in early 2021 to include those with at least a 20 pack-year smoking history, decreased from the original 30 pack-year criterion.^28^ There was no clear effect of this change on LDCT screening rates in Figure 2.

### Limitations

A major challenge for any study using administrative claims to determine cancer screening rates is the inability to determine a true denominator. With both the mammography and LDCT cohorts, we made no attempt to remove from the cohort those enrollees who might have had an indication other than screening, for example, evaluation of a breast mass found by self examination. We took this approach because routine ambulatory care was disrupted during the pandemic,^4^ which would result in lower rates of documentation of indications and affect estimates of screening. Including all age-appropriate enrollees avoids that issue while somewhat overestimating true screening rates. This is particularly an issue with LDCT, where we generated rates for an age-appropriate population but could not determine which enrollees satisfied the other criteria for LDCT screening, in particular, smoking status. The assumption is that the temporal pattern in rates generated using the larger Medicare population is indicative of the pattern of LDCT screening in the population that meets screening criteria. One reason this might not be the case is if there was selective attrition of smokers during the pandemic. In that case, an unchanged LDCT rate in the smoking population might produce a decreased LDCT rate for the general population. However, the excess COVID-19 deaths among smokers was a small proportion of all smokers, and would have only a minor effect on the analyses. Similarly, differences in LDCT screening rates by enrollee characteristics might reflect differences in screening eligibility by those characteristics rather than true disparities in screening.

Another limitation is that our cohorts were restricted to fee-for-service Medicare recipients. Over the period studied (January 17 to July 22), the proportion of all Medicare enrollees under fee for service declined (eTable 3 in Supplement 1). In addition, HMO enrollees may have had different patterns of screening. Also, it is important to note that Medicare enrollees younger than 65 years are a special population, comprised of those with disabilities, end-stage renal disease or amyotrophic lateral sclerosis. Thus, the results for that cohort do not reflect the general population. In analyses stratified by those younger than 65 years vs those aged 65 years or older, the overall patterns were the same, but the younger enrollees had somewhat greater depression of screening rates during March 20 to February 21 and March 21 to February 22.

Current breast cancer screening recommendations are for biannual mammography for women at average risk.^29^ That means the drop in rates seen in any year cannot be directly translated to deficits in mammography screening.^9^

## Conclusions

Delays in cancer screening can contribute to delays in diagnosis and increased deaths from cancer.^1,10,27,30^ Ongoing efforts to raise vaccination rates may gradually lower concerns among enrollees about infection risk during medical encounters. Also, screening for some cancers, including colorectal and cervical cancer, can be initially conducted via home testing, and there is some evidence that such testing increased during the pandemic.^9,27^ More widespread recognition of the slow recovery from pandemic-related deficits in cancer screening should lead to more focused efforts to remedy the deficits.

## Notes

Supplement 1. **eFigure 1.** Cohort selection for mammogram rates **eFigure 2.** Cohort selection for low dose computed tomography rates **eTable 1.** Patient characteristics for the mammography cohort for 2017 to 2021 **eTable 2.** Patient characteristics for the low dose computed tomography (LDCT) cohort from 2018 (March 2017-Feb 2018) to 2021 (March 2021-Feb 2022) **eTable 3.** Percentage of Medicare enrollees enrolled in HMO during 2017 through 2022, using 100% Medicare data in January for each year, by enrollee characteristics **eTable 4.** Logistic regression model using cohorts from 2017-2019 to predict the expected mammogram rate in March 2020 to February 2021 **eTable 5.** Logistic regression model using cohorts from 2017-2019 to predict the expected LDCT rate in March 2020 to February 2021 **eTable 6.** Stratified analysis for beneficiaries aged <65 **eTable 7.** Stratified analysis for beneficiaries aged ≥65 **eTable 8.** Stratified analysis for beneficiaries aged <65 **eTable 9.** Stratified analysis for beneficiaries aged ≥65 **eTable 10.** Stratified analysis for Medicaid beneficiaries **eTable 11.** Stratified analysis for non-Medicaid beneficiaries **eTable 12.** Stratified analysis for Medicaid beneficiaries **eTable 13.** Stratified analysis for non-Medicaid beneficiaries

Supplement 2.
Data Sharing Statement
