# Supplementary material for: Breast and Lung Cancer Screening Among Medicare Enrollees During the COVID-19 Pandemic
Source: JAMA Netw Open. 2023 Feb 3;6(2):e2255589. doi: 10.1001/jamanetworkopen.2022.55589 (PMC9898823; doi:10.1001/jamanetworkopen.2022.55589)
Supplement: Supplement 1. — eFigure 1. Cohort selection for mammogram rates eFigure 2. Cohort selection for low dose computed tomography rates eTable 1. Patient characteristics for the mammography cohort for 2017 to 2021 eTable 2. Patient characteristics for the low dose computed tomography (LDCT) cohort from 2018 (March 2017-Feb 2018) to 2021 (March 2021-Feb 2022) eTable 3. Percentage of Medicare enrollees enrolled in HMO during 2017 through 2022, using 100% Medicare data in January for each year, by enrollee characteristics eTable 4. Logistic regression model using cohorts from 2017-2019 to predict the expected mammogram rate in March 2020 to February 2021 eTable 5. Logistic regression model using cohorts from 2017-2019 to predict the expected LDCT rate in March 2020 to February 2021 eTable 6. Stratified analysis for beneficiaries aged <65 eTable 7. Stratified analysis for beneficiaries aged ≥65 eTable 8. Stratified analysis for beneficiaries aged <65 eTable 9. Stratified analysis for beneficiaries aged ≥65 eTable 10. Stratified analysis for Medicaid beneficiaries eTable 11. Stratified analysis for non-Medicaid beneficiaries eTable 12. Stratified analysis for Medicaid beneficiaries eTable 13. Stratified analysis for non-Medicaid beneficiaries [file jamanetwopen-e2255589-s001.pdf]

Supplemental Online Content

Doan C, Li S, Goodwin JS. Breast and lung cancer screening among Medicare enrollees during the COVID-19 pandemic. *JAMA Network Open*. 2023;6(2):e2255589.
doi:10.1001/jamanetworkopen.2022.55589

eFigure 1. Cohort selection for mammogram rates

eFigure 2. Cohort selection for low dose computed tomography rates

eTable 1. Patient characteristics for the mammography cohort for 2017 to 2021

eTable 2. Patient characteristics for the low dose computed tomography (LDCT) cohort from 2018 (March 2017-Feb 2018) to 2021 (March 2021-Feb 2022)

eTable 3. Percentage of Medicare enrollees enrolled in HMO during 2017 through 2022, using 100% Medicare data in January for each year, by enrollee characteristics

eTable 4. Logistic regression model using cohorts from 2017-2019 to predict the expected mammogram rate in March 2020 to February 2021

eTable 5. Logistic regression model using cohorts from 2017-2019 to predict the expected LDCT rate in March 2020 to February 2021

eTable 6. Stratified analysis for beneficiaries aged <65

eTable 7. Stratified analysis for beneficiaries aged ≥65

eTable 8. Stratified analysis for beneficiaries aged <65

eTable 9. Stratified analysis for beneficiaries aged ≥65

eTable 10. Stratified analysis for Medicaid beneficiaries

eTable 11. Stratified analysis for non-Medicaid beneficiaries

eTable 12. Stratified analysis for Medicaid beneficiaries

eTable 13. Stratified analysis for non-Medicaid beneficiaries

This supplemental material has been provided by the authors to give readers additional information about their work.

© 2023 Doan C et al. *JAMA Network Open.*

Supplementary eFigure 1. Cohort selection for mammogram rates.

1) Choose all female beneficiaries who alive as of March 1st in each year, age between 50 and 74.
N=4,105,930 (100%) 2017
N=4,241,886 (100%) 2018
N=4,348,051 (100%) 2019
N=4,449,120 (100%) 2020
N=4,525,374 (100%) 2021

↓

2) Keep the beneficiaries who have part A and B and no HMO during 1 year prior to March 1st and 1 year after
N=1,710,748 (41.67%) 2017
N=1,708,973 (40.29%) 2018
N=1,695,332 (38.99%) 2019
N=1,664,249 (37.41%) 2020
N=1,609,417 (35.56%) 2021

© 2023 Doan C et al. *JAMA Network Open.*

Supplementary eFigure 2. Cohort selection for low dose computed tomography rates. HMO: health maintenance organization.

1) Choose all beneficiaries who alive as of March 1st in each year, age between 55 and 79.
N=8,449,695 (100%) 2017
N=8,784,886 (100%) 2018
N=9,092,209 (100%) 2019
N=9,374,593 (100%) 2020
N=9,584,406 (100%) 2021

↓

2) Keep the beneficiaries who have part A and B and no HMO during 1 year prior to March 1st and 1 year after
N=3,875,754 (45.87%) 2017
N=3,898,228 (44.37%) 2018
N=3,883,897 (42.72%) 2019
N=3,835,272 (40.91%) 2020
N=3,719,971 (38.81%) 2021

© 2023 Doan C et al. *JAMA Network Open.*

Supplementary eTable 1. Patient characteristics for the mammography cohort for 2017 to 2021.

	March 2017-Feb 2018	March 2018-Feb 2019	March 2019-Feb 2020	March 2020-Feb 2021	March 2021-Feb 2022
All	1,710,748	1,708,973	1,695,332	1,664,249	1,609,417
Age, years					
50-65	331,680 (19.39%)	313,215 (18.33%)	287,082 (16.93%)	256,399 (15.41%)	223,032 (13.86%)
66-70	857,556 (50.13%)	853,145 (49.92%)	838,723 (49.47%)	817,991 (49.15%)	778,436 (48.37%)
71-74	521,512 (30.48%)	542,613 (31.75%)	569,527 (33.59%)	589,859 (35.44%)	607,949 (37.77%)
Race					
Black	163,876 (9.58%)	158,447 (9.27%)	149,827 (8.84%)	137,413 (8.26%)	122,910 (7.64%)
Hispanic	95,903 (5.61%)	94,951 (5.56%)	92,534 (5.46%)	89,732 (5.39%)	84,877 (5.27%)
White	1,359,092 (79.44%)	1,357,595 (79.44%)	1,349,745 (79.62%)	1,331,378 (80.00%)	1,295,767 (80.51%)
Other[a]	91,877 (5.37%)	97,980 (5.73%)	103,226 (6.09%)	105,726 (6.35%)	105,863 (6.58%)
Medicaid					
No	1,389,689 (81.23%)	1,404,176 (82.16%)	1,414,547 (83.44%)	1,410,852 (81.77%)	1,385,133 (86.06%)
Yes	321,059 (18.77%)	304,797 (17.84%)	280,785 (16.56%)	253,397 (15.23%)	224,284 (13.94%)
PCP					
No	1,134,367 (66.31%)	1,158,765 (67.80%)	1,177,032 (69.43%)	1,179,111 (70.85%)	21,241,617 (77.15%)
Yes	576,381 (33.69%)	550,208 (32.20%)	518,300 (30.57%)	485,138 (29.15%)	367,800 (22.85%)[b]
AHRQ comorbidity score					
<0	587,968 (34.37%)	607,827 (35.22%)	607,345 (35.82%)	599,865 (36.04%)	530,126 (32.94%)
0	750,489 (43.87%)	736,962 (43.12%)	723,740 (42.69%)	707,071 (42.49%)	753,420 (46.81%)
>=	372,291 (21.76%)	370,184 (21.66%)	364,247 (21.49%)	357,313 (21.47%)	325,871 (20.25%)

PCP: Primary care provider; AHRQ: Agency for Healthcare Research and Quality.

[a]Other race incudes American Indian/Alaskan native, Asian/Pacific Islanders, and other.

[b]The decrease in percentage of subjects with an identifiable PCP in the 2021 cohort may be an artifact of the method of assessing PCPs, which is to examine the prior 12 months of data. For the 2021 cohorts, the prior 12 months included the first 10 months of the COVID pandemic, when regular ambulatory care was substantially disrupted.[4]

© 2023 Doan C et al. *JAMA Network Open.*

Supplementary eTable 2. Patient characteristics for the LDCT cohort from 2018 (March 2017-Feb 2018) to 2021 (March 2021-Feb 2022).

	March 2017-Feb 2018	March 2018-Feb 2019	March 2019-Feb 2020	March 2020-Feb 2021	March 2021-Feb 2022
All	3,875,754	3,898,228	3,883,897	3,835,272	3,719,971
Age, years					
55-65	510,086 (13.16%)	489,261 (12.55%)	454,202 (11.69%)	410,365 (10.70%)	359,436 (9.66%)
66-70	1,540,109 (39.74%)	1,525,813 (39.14%)	1,493,768 (38.46%)	1,453,807 (37.91%)	1,385,791 (37.25%)
71-75	1,137,032 (29.34%)	1,194,865 (30.65%)	1,231,248 (31.70%)	1,256,900 (32.77%)	1,271,074 (34.17%)
76-79	688,527 (17.76%)	688,289 (17.66%)	704,679 (18.14%)	714,200 (18.62%)	703,670 (18.92%)
Sex					
Male	1,779,908 (45.92%)	1,787,343 (45.85%)	1,776,643 (45.74%)	1,750,617 (45.65%)	1,692,459 (45.50%)
Female	2,095,846 (54.08%)	2,110,885 (54.15%)	2,107,254 (54.26%)	2,084,655 (54.35%)	2,027,512 (54.50%)
Race					
Black	323,766 (8.35%)	316,623 (8.12%)	302,473 (7.79%)	280,717 (7.32%)	251,921 (6.77%)
Hispanic	203,321 (5.25%)	202,709 (5.20%)	198,492 (5.11%)	194,044 (5.06%)	184,225 (4.95%)
White	3,132,154 (80.81%)	3,147,797 (80.75%)	3,140,012 (80.85%)	3,110,315 (81.10%)	3,032,100 (81.51%)
Other[a]	216,513 (5.59%)	231,099 (5.93%)	242,920 (6.25%)	250,196 (6.52%)	251,725 (6.77%)
Medicaid					
No	3,313,064 (85.48%)	3,354,348 (86.05%)	3,372,481 (86.83%)	3,362,908 (87.68%)	3,295,732 (88.60%)
Yes	562,690 (14.52%)	543,880 (13.95%)	511,416 (13.17%)	472,364 (12.32%)	424,239 (11.40%)
PCP					
No	2,590,533 (66.84%)	2,656,190 (68.14%)	2,700,860 (69.54%)	2,712,106 (70.71%)	2,864,461 (77.00%)
Yes	1,284,821 (33.15%)	1,242,038 (31.86%)	1,183,037 (30.46%)	1,123,166 (29.29%)	855,510 (23.00%)[b]
AHRQ comorbidity score					
<0	1,059,573 (27.34%)	1,101,063 (28.25%)	1,130,634 (29.11%)	1,129,729 (29.46%)	1,005,999 (27.04%)
0	1,794,636 (46.30%)	1,761,198 (45.18%)	1,723,998 (44.39%)	1,679,954 (43.80%)	1,774,451 (47.70%)
>0	1,021,545 (26.36%)	1,032,967 (26.50%)	1,029,265 (26.50%)	1,025,589 (26.74%)	939,521 (25.26%)

LDCT: low dose computed tomography; PCP: Primary care provider; AHRQ: Agency for Healthcare Research and Quality.
aOther race incudes American Indian/Alaskan native, Asian/Pacific Islanders, and other.
[b]The decrease in percentage of subjects with an identifiable PCP in the 2021 cohort may be an artifact of the method of assessing PCPs, which is to examine the prior 12 months of data. For the 2021 cohorts, the prior 12 months included the first 10 months of the COVID pandemic, when regular ambulatory care was substantially disrupted.[4]

© 2023 Doan C et al. *JAMA Network Open.*

Supplementary eTable 3. Percentage of Medicare enrollees enrolled in a Health Maintenance Organization (HMO) during 2017 through 2021, using 100% Medicare data in January for each year, by enrollee characteristics.

	Percentage of HMO enrollment (%)				
	2017	2018	2019	2020	2021
**All**	31.82	33.41	34.98	37.37	40.32
**Age**					
<65	21.36	22.93	24.86	27.85	31.06
>=65	34.54	36.05	37.44	39.56	42.36
**Sex**					
Male	30.31	31.82	33.25	35.57	38.50
Female	33.09	34.76	36.45	38.89	41.86
**Medicaid**					
No	31.55	32.91	34.09	36.01	38.48
Yes	33.02	35.72	39.15	43.77	48.88
**Race**					
Black	29.55	30.98	32.35	34.53	37.30
Hispanic	36.15	38.78	41.34	44.90	49.53
White	46.41	47.97	49.76	52.25	54.77
Other[a]	29.73	31.23	32.84	35.06	37.73

[a]Other race incudes American Indian/Alaskan native, Asian/Pacific Islanders, and other.

© 2023 Doan C et al. *JAMA Network Open.*

Supplementary eTable 4. Logistic regression model using cohorts from 2017-2019 to predict the expected mammogram rate in March 2020 to February 2021. The 95% confidence interval (CI) for expected rate was calculated using the bootstrap method with 30 runs.

	**Expected Mammogram rate (95% CI)**	**Observed Mammogram rate**	**O/E ratio (95% CI)**
All	47.85% (47.82%, 47.88%)	39.53%	0.83 (0.82, 0.83)
Age, years			
50-65	36.09% (36.06%, 36.12%)	27.50%	0.76 (0.76, 0.76)
66-70	50.71% (50.68%, 50.74%)	42.31%	0.83 (0.83, 0.84)
71-74	49.00% (48.96%, 83.55%)	40.91%	0.83 (0.83, 0.84)
Race			
Black	45.40% (45.36%, 45.45%)	37.15%	0.82 (0.81, 0.82)
Hispanic	39.17% (39.11%, 39.23%)	29.29%	0.75 (0.74, 0.75)
White	48.89% (48.86%, 48.92%)	40.83%	0.84 (0.83, 0.84)
Other[a]	45.31% (45.26%, 45.35%)	34.96%	0.77 (0.77, 0.78)
Medicaid			
No	50.54% (50.51%, 50.57%)	42.45%	0.84 (0.83, 0.84)
Yes	32.88% (32.85%, 32.91%)	23.26%	0.71 (0.70, 0.71)
PCP			
No	45.93% (45.89%, 45.96%)	37.72%	0.82 (0.82, 0.83)
Yes	52.53% (52.49%, 52.57%)	43.94%	0.84 (0.84, 0.84)
Comorbidity score			
<0	51.24% (51.21%, 51.28%)	42.60%	0.83 (0.83, 0.83)
0	46.90% (46.86%, 46.93%)	38.26%	0.82 (0.82, 0.83)
>0	44.05% (44.01%, 44.09%)	36.89%	0.84 (0.84, 0.85)

O/E: observed/expected; PCP: Primary care provider.

[a]Other race incudes American Indian/Alaskan native, Asian/Pacific Islanders, and other.

© 2023 Doan C et al. *JAMA Network Open.*

Supplementary eTable 5. Logistic regression model using cohorts from 2017-2019 to predict the expected LDCT rate in March 2020 to February 2021. The 95% confidence interval (CI) for expected rate was calculated using the bootstrap method with 30 runs.

	Expected LDCT rate per 1,000,000 (95% CI)	Observed LDCT rate per 1,000,000	O/E ratio (95% CI)
All	16,436 (16,402, 16,471)	11,411	0.69 (0.69, 0.70)
Age, years			
55-65	28,832 (28,986, 28,965)	19,690	0.68 (0.68, 0.69)
66-70	19,380 (19,349, 19,424)	13,383	0.69 (0.68, 0.69)
71-75	15,428 (15,388, 15,467)	10,670	0.69 (0.68, 0.69)
76-79	5,097 (5,062, 5,131)	3,944	0.77 (0.77, 0.78)
Sex			
Male	18,214 (18,161, 18,266)	12,790	0.70 (0.70, 0.71)
Female	14,944 (14,910, 14,977)	10,253	0.69 (0.68, 0.69)
Race			
Black	11,945 (11,893, 11,998)	7,983	0.67 (0.66, 0.67)
Hispanic	6,787 (6,720, 6,854)	4,174	0.62 (0.61, 0.62)
White	17,955 (17,915, 17,996)	12,513	0.70 (0.69, 0.70)
Other[a]	10,074 (10,030, 10,145)	7,178	0.71 (0.71, 0.72)
Medicaid			
No	15,520 (15,487, 15,553)	10,886	0.70 (0.70, 0.71)
Yes	22,958 (22,885, 23,030)	15,154	0.66 (0.65, 0.66)
PCP			
No	15,140 (15,103, 15,176)	10,480	0.69 (0.69, 0.70)
Yes	19,567 (19,510, 19,624)	13,632	0.70 (0.69, 0.70)
Comorbidity score			
<0	18,768 (18,722, 18,814)	13,133	0.70 (0.69, 0.70)
0	11,229 (11,193, 11,265)	7,234	0.64 (0.64, 0.65)
>0	22,397 (22,336, 22,458)	16,357	0.73 (0.73, 0.73)

LDCT: low dose computed tomography; O/E: observed/expected; PCP: PCP: Primary care provider.
[a]Other race includes American Indian/Alaskan native, Asian/Pacific Islanders, and other.

© 2023 Doan C et al. *JAMA Network Open.*

Supplementary eTable 6. Expected and observed mammogram rates for enrollees aged ≥50 to <65 years for 3/20 to 2/21 and 3/21 to 2/22 from a prediction model using 2017-2019 cohorts, with a linear probability model for each sub-cohort, with year as the only variable in the models.

	3/20 to 2/21			3/21 to 2/22		
Enrollees (<65)	Expected Mammogram rate, % (95% CI)	Observed Mammogram rate, %	O/E ratio (95% CI)	Expected Mammogram rate, % (95% CI)	Observed Mammogram rate, %	O/E ratio (95% CI)
All	33.54 (33.26-33.82)	26.09	0.78 (0.77-0.78)	33.85 (33.45-34.25)	31.22	0.92 (0.91-0.93)
Race						
Black	37.01 (36.33-37.68)	29.62	0.80 (0.79-0.82)	37.24 (36.28-38.20)	34.24	0.92 (0.90-0.94)
Hispanic	35.12 (34.14-36.10)	26.44	0.75 (0.73-0.77)	35.48 (34.08-36.87)	31.41	0.89 (0.85-0.92)
White	32.55 (32.21-32.88)	25.36	0.78 (0.77-0.79)	32.90 (32.42-33.37)	30.60	0.93 (0.89-0.92)
Other[a]	32.29 (30.87-33.70)	22.92	0.71 (0.68-0.74)	32.54 (30.51-34.56)	29.20	0.90 (0.84-0.96)
Medicaid						
No	33.87 (33.46-34.27)	26.99	0.80 (0.79-0.81)	34.27 (33.69-34.84)	32.22	0.94 (0.92-0.96)
Yes	33.24 (32.85-33.62)	25.22	0.76 (0.75-0.77)	33.46 (32.91-34.02)	30.21	0.90 (0.89-0.92)
PCP						
No	30.59 (30.25-30.92)	23.56	0.77 (0.76-0.79)	30.84 (30.36-31.32)	29.14	0.95 (0.93-0.96)
Yes	40.23 (39.72-40.73)	31.94	0.79 (0.78-0.80)	41.11 (40.39-41.83)	37.58	0.91 (0.90-0.93)
Comorbidity score						
<0	38.18 (37.77-38.59)	29.87	0.78 (0.77-0.79)	38.76 (38.17-39.35)	35.81	0.92 (0.91-0.94)
0	26.15 (25.63-26.68)	19.74	0.75 (0.74-0.77)	25.85 (25.10-26.59)	24.67	0.95 (0.93-0.98)
>0	31.78 (31.24-32.32)	25.09	0.79 (0.78-0.80)	32.00 (31.22-32.77)	30.33	0.95 (0.93-0.97)

[a]Other includes Asian/pacific islander, American Indian/Alaska native or others.
O/E: observed/expected; CI: Confidence Interval; PCP: Primary care provider.

© 2023 Doan C et al. *JAMA Network Open.*

Supplementary eTable 7. Stratified analysis for beneficiaries aged >=65. Expected and observed mammogram rates for enrollees aged $\geq$65 years for 3/20 to 2/21 and 3/21 to 2/22 from a prediction model using 2017-2019 cohorts, with a linear probability model for each sub-cohort, with year as the only variable in the models.

Age 65-74	Expected Mammogram rate, % (95% CI)	Observed Mammogram rate, %	O/E ratio (95% CI)	Expected Mammogram rate, % (95% CI)	Observed Mammogram rate, %	O/E ratio (95% CI)
All	50.03 (49.90-50.15)	41.56	0.83 (0.82-0.83)	51.10 (50.92-51.28)	49.22	0.96 (0.96-0.97)
Race						
Black	47.54 (47.09-48.00)	40.02	0.84 (0.83-0.85)	48.53 (47.87-49.18)	47.05	0.97 (0.96-0.98)
Hispanic	39.15 (38.61-39.69)	30.05	0.77 (0.76-0.78)	39.82 (39.04-40.59)	37.40	0.94 (0.92-0.96)
White	51.19 (51.06-51.33)	42.84	0.84 (0.83-0.84)	52.30 (52.10-52.50)	50.36	0.96 (0.96-0.97)
Other[a]	46.56 (46.07-47.05)	36.04	0.77 (0.77-0.78)	47.71 (47.00-48.43)	46.01	0.97 (0.95-0.98)
Medicaid						
No	52.23 (52.10-52.36)	43.73	0.84 (0.83-0.84)	53.27 (53.08-53.46)	51.39	0.96 (0.96-0.97)
Yes	30.31 (29.96-30.65)	21.73	0.72 (0.71-0.73)	30.51 (30.02-31.01)	27.68	0.91 (0.89-0.92)
PCP						
No	48.17 (48.02-48.32)	39.83	0.83 (0.82-0.83)	49.31 (49.09-49.52)	47.94	0.97 (0.97-0.98)
Yes	54.59 (54.36-54.81)	45.82	0.84 (0.83-0.84)	55.81 (55.49-56.14)	53.61	0.96 (0.95-0.97)
Comorbidity score						
<0	54.56 (54.34-54.78)	45.36	0.83 (0.83-0.84)	55.86 (55.56-56.18)	53.62	0.96 (0.95-0.97)
0	48.38 (48.20-48.56)	39.82	0.82 (0.82-0.83)	49.23 (48.97-49.50)	47.71	0.97 (0.96-0.97)
>0	46.06 (45.78-46.33)	39.12	0.85 (0.84-0.85)	46.99 (46.60-47.38)	46.02	0.98 (0.97-0.99)

[a]Other includes Asian/Pacific Islander, American Indian/Alaska native, or others.
CI: confidence interval; O/E ratio: observed/expected ratio; PCP: primary care provider.

© 2023 Doan C et al. *JAMA Network Open.*

Supplementary eTable 8. Stratified analysis for beneficiaries aged <65. Expected and observed LDCT rates for 3/20 to 2/21 and 3/21 to 2/22 from a prediction model using 2017-2019 cohorts, with a linear probability model for each sub-cohort, with year as the only variable in the models.

Age <65	**3/20 to 2/21**			**3/21 to 2/22**		
	Expected LDCT rate per 1,000,000 (95% CI)	Observed LDCT rate per 1,000,000	O/E ratio (95% CI)	Expected LDCT rate per 1,000,000 (95% CI)	Observed LDCT rate	O/E ratio (95% CI)
All	27,391 (26,727-28,055)	20,246	0.74 (0.72-0.76)	32,573 (31,640-33,506)	26,403	0.81 (0.79-0.83)
Sex						
Male	25,757 (24,851-26,663)	18,908	0.73 (0.71-0.86)	30,594 (29,322-31,866)	24,874	0.81 (0.78-0.85)
Female	29,083 (28,107-30,059)	21,654	0.75 (0.72-0.77)	34,629 (33,357-35,901)	28,024	0.81 (0.78-0.84)
Race						
Black	13,927 (12,737-15,118)	9,603	0.69 (0.64-0.75)	16,129 (14,453-17,805)	14,485	0.90 (0.81-1.02)
Hispanic	9,140 (7,743-10,537)	7,114	0.78 (0.68-0.92)	10,704 (8,734-12,674)	9,510	0.89 (0.75-1.01)
White	33,381 (32,515-34,247)	24,785	0.74 (0.72-0.76)	39,812 (38,595-41,029)	31,689	0.80 (0.77-0.82)
Other[a]	15,454 (12,896-18,012)	10,753	0.70 (0.60-0.83)	18,418 (14,821-22,015)	15,053	0.82 (0.68-1.02)
Medicaid						
No	23,769 (22,926-24,612)	17,770	0.75 (0.72-0.78)	28,290 (27,108-29,472)	23,690	0.84 (0.80-0.87)
Yes	31,724 (30,666-32,782)	23,281	0.73 (0.71-0.76)	37,700 (36,216-39,184)	29,762	0.79 (0.76-0.82)
PCP						
No	25,003 (24,250-25,756)	17,892	0.72 (0.69-0.74)	29,863 (28,808-30,918)	24,644	0.83 (0.80-0.86)
Yes	33,441 (32,087-34,795)	26,478	0.79 (0.76-0.83)	39,622 (37,727-41,517)	32,633	0.82 (0.79-0.86)
Comorbidity score						
<0	33,417 (32,266-34,568)	25,027	0.75 (0.72-0.78)	39,834 (38,219-41,449)	33,152	0.83 (0.80-0.87)
0	17,030 (16,081-17,987)	11,708	0.69 (0.65-0.73)	20,111 (18,772-21,450)	17,514	0.87 (0.82-0.93)
>0	29,917 (28,609-31,225)	22,642	0.76 (0.73-0.79)	35,451 (33,615-37,287)	28,179	0.79 (0.76-0.84)

[a]Other includes Asian/pacific islander, American Indian/Alaska native or others
O/E: observed/expected; CI: Confidence Interval; PCP: Primary care provider.

© 2023 Doan C et al. *JAMA Network Open.*

Supplementary eTable 9. Stratified analysis for beneficiary aged >=65. Expected and observed low dose computed tomography (LDCT) rates for 3/20 to 2/21 and 3/21 to 2/22 from a prediction model using 2017-2019 cohorts, with a linear probability model for each sub-cohort, with year as the only variable in the models.

Age >=65	Expected LDCT rate per 1,000,000 (95% CI)	Observed LDCT rate per 1,000,000	O/E ratio (95% CI)	Exp ected LDCT rate per 1,000,000 (95% CI)	Observed LDCT rate	O/E ratio (95% CI)
All	13,628 (13,467-13,787)	10,553	0.77 (0.77-0.78)	16,132 (15,909-16,356)	14,237	0.88 (0.87-0.89)
Sex						
Male	15,449 (15,200-15,698)	12,115	0.78 (0.77-0.80	18,292 (17,941-18,643)	16,130	0.88 (0.87-0.90)
Female	12,133 (12,335-11,931)	9,270	0.76 (0.75-0.78)	14,345 (14,629-14,061)	12,690	0.88 (0.87-0.90)
Race						
Black	9,894 (9,382-10,406)	7,586	0.77 (0.73-0.81)	11,746 (11,025-12,467)	10,582	0.90 (0.85-0.96)
Hispanic	5,301 (4,843-5,759)	3,662	0.69 (0.64-0.76)	6,228 (5,581-6,874)	5,884	0.94 (0.86-1.05)
White	14,787 (14,605-14,969)	12,176	0.82 (0.81-0.83)	17,512 (17,255-17,769)	15,354	0.88 (0.86-0.90)
Other[a]	9,028 (8,522-9,534)	6,972	0.77 (0.73-0.82)	10,747 (10,032-11,462)	9,762	0.91 (0.85-0.97)
Medicaid						
No	13,387 (13,222-13,552)	10,480	0.78 (0.77-0.79)	15,841 (15,608-16,074)	14,070	0.89 (0.88-0.90)
Yes	15,883 (15,344-16,422)	11,278	0.71 (0.69-0.74)	18,904 (18,147-19,661)	16,026	0.85 (0.82-0.88)
PCP						
No	12,601 (12,419-12,783)	9,752	0.77 (0.76-0.79)	14,968 (14,711-15,225)	13,582	0.91 (0.85-0.92)
Yes	16,013 (15,705-16,321)	12,471	0.78 (0.76-0.79)	18,902 (18,469-19,335)	16,420	0.87 (0.85-0.89)
Comorbidity score						
<0	14,687 (14,375-14,999)	11,478	0.78 (0.77-0.80)	17,392 (16,951-17,833)	15,776	0.91 (0.88-0.93)
0	9,520 (9,324-9,716)	6,937	0.73 (0.71-0.74)	11,217 (10,941-11,493)	10,155	0.91 (0.88-0.93)
>0	19,574 (19,206-19,942)	15,703	0.80 (0.79-0.82)	23,157 (22,638-23,676)	20,584	0.89 (0.87-0.91)

[a]Other includes Asian/Pacific Islander, American Indian/Alaska native, or others.
CI: confidence interval; O/E ratio: observed/expected ratio; PCP: primary care provider.

© 2023 Doan C et al. *JAMA Network Open.*

Supplementary eTable 10. Stratified analysis for Medicaid beneficiaries. Expected and observed mammogram rates for 3/20 to 2/21 and 3/21 to 2/22 from a prediction model using 2017-2019 cohorts, with a linear probability model for each sub-cohort, with year as the only variable in the models.

	**3/20 to 2/21**			**3/21 to 2/22**		
Medicaid Enrollees	Expected Mammogram rate, % (95% CI)	Observed Mammogram rate, %	O/E ratio (95% CI)	Expected Mammogram rate, % (95% CI)	Observed Mammogram rate, %	O/E ratio (95% CI)
All	31.61 (31.35-31.87)	23.26	0.74 (0.73-0.74)	31.80 (31.43-32.17)	28.74	0.90 (0.89-0.91)
Age, years						
50-65	33.14 (32.77-33.51)	25.18	0.76 (0.75-0.77)	33.33 (32.79-33.86)	30.17	0.91 (0.89-0.92)
66-74	30.20 (29.85-30.56)	21.54	0.71 (0.70-0.72)	30.45 (29.94-30.96)	27.55	0.90 (0.89-0.92)
Race						
Black	34.66 (34.05-35.28)	26.91	0.78 (0.76-0.79)	34.60 (33.72-35.48)	31.64	0.91 (0.89-0.94)
Hispanic	34.95 (34.22-35.67)	24.77	0.71 (0.69-0.72)	35.13 (34.08-36.17)	31.14	0.89 (0.86-0.91)
White	29.88 (29.55-30.21)	22.26	0.74 (0.74-0.75)	30.14 (29.68-30.61)	27.18	0.90 (0.89-0.92)
Other[a]	32.06 (31.16-32.97)	20.44	0.64 (0.62-0.66)	32.28 (30.98-33.58)	29.82	0.92 (0.89-0.96)
PCP						
No	28.67 (28.36-28.98)	20.78	0.72 (0.72-0.73)	28.85 (28.41-29.30)	26.66	0.92 (0.91-0.94)
Yes	38.03 (37.57-38.49)	28.75	0.76 (0.75-0.77)	38.70 (38.04-39.35)	35.07	0.91 (0.89-0.92)
Comorbidity score						
<0	36.61 (36.21-37.01)	27.19	0.74 (0.73-0.75)	37.16 (36.58-37.73)	33.52	0.90 (0.89-0.92)
0	26.87 (26.38-27.36)	18.76	0.70 (0.69-0.71)	26.55 (25.85-27.24)	24.92	0.94 (0.91-0.96)
>0	28.17 (27.71-28.63)	21.32	0.76 (0.74-0.77)	28.18 (27.51-28.83)	25.96	0.92 (0.90-0.94)

[a]Other includes Asian/Pacific Islander, American Indian/Alaska native, or others.
CI: confidence interval; O/E ratio: observed/expected ratio; PCP: primary care provider.

© 2023 Doan C et al. *JAMA Network Open.*

Supplementary eTable 11. Stratified analysis for non-Medicaid beneficiaries. Expected and observed mammogram rates for 3/20 to 2/21 and 3/21 to 2/22 from a prediction model using 2017-2019 cohorts, with a linear probability model for each sub-cohort, with year as the only variable in the models.

Non-Medicaid Enrollees	Expected Mammogram rate, % (95% CI)	Observed Mammogram rate, %	O/E ratio (95% CI)	Expected Mammogram rate, % (95% CI)	Observed Mammogram rate, %	O/E ratio (95% CI)
All	50.80 (50.68-50.93)	42.45	0.84 (0.83-0.84)	51.90 (51.72-52.08)	50.08	0.96 (0.96-0.97)
Age, years						
50-65	36.94 (36.57-37.30)	29.54	0.80 (0.79-0.81)	37.54 (37.02-38.07)	35.36	0.94 (0.93-0.96)
66-74	52.33 (52.20-52.46)	43.84	0.84 (0.83-0.84)	53.37 (53.17-53.56)	51.49	0.96 (0.96-0.97)
Race						
Black	49.49 (49.01-49.96)	42.06	0.85 (0.84-0.86)	50.60 (49.92-51.29)	49.03	0.97 (0.96-0.98)
Hispanic	40.47 (39.85-41.10)	32.22	0.80 (0.78-0.81)	41.32 (40.42-42.22)	39.32	0.95 (0.93-0.97)
White	51.52 (51.38-51.66)	43.20	0.84 (0.84-0.84)	52.62 (52.42-52.82)	50.75	0.96 (0.96-0.97)
Other[a]	49.05 (48.51-49.59)	38.95	0.79 (0.78-0.80)	50.27 (49.49-51.05)	48.69	0.97 (0.95-0.98)
PCP						
No	48.91 (48.76-49.06)	40.66	0.83 (0.83-0.83)	50.07 (49.85-50.29)	48.79	0.97 (0.97-0.98)
Yes	55.55 (55.27-55.73)	46.88	0.84 (0.84-0.85)	56.74 (56.41-57.06)	54.53	0.96 (0.96-0.97)
Comorbidity score						
<0	55.12 (54.90-55.34)	46.16	0.84 (0.83-0.84)	56.43 (56.11-56.74)	54.32	0.96 (0.96-0.97)
0	48.68 (48.50-48.87)	40.26	0.83 (0.82-0.83)	49.57 (49.31-49.84)	48.17	0.97 (0.97-0.98)
>0	48.04 (47.76-48.32)	41.04	0.85 (0.85-0.86)	49.00 (48.60-49.41)	48.02	0.98 (0.97-0.99)

[a]Other includes Asian/Pacific Islander, American Indian/Alaska native, or others.
CI: confidence interval; O/E ratio: observed/expected ratio; PCP: primary care provider.

© 2023 Doan C et al. *JAMA Network Open.*

Supplementary eTable 12. Stratified analysis for Medicaid beneficiaries. Expected and observed low dose computed tomography (LDCT) rates for 3/20 to 2/21 and 3/21 to 2/22 from a prediction model using 2017-2019 cohorts, with a linear probability model for each sub-cohort, with year as the only variable in the models.

Medicaid	**3/20 to 2/21**			**3/21 to 2/22**		
	Expected LDCT rate per 1,000,000 (95% CI)	Observed LDCT rate per 1,000,000	O/E ratio (95% CI)	Expected LDCT rate per 1,000,000 (95% CI)	Observed LDCT rate	O/E ratio (95% CI)
All	21,173 (20,668-21,679)	15,154	0.72 (0.70-0.73)	25,144 (24,435-25,854)	20,288	0.81 (0.78-0.83)
Age, years						
50-65	31,464 (30,455-32,473)	23,096	0.73 (0.71-0.76)	37,411 (36,001-38,821)	29,558	0.79 (0.76-0.82)
66-74	15,192 (14,651-15,733)	10,765	0.71 (0.68-0.74)	18,061 (17,301-18,821)	15,431	0.85 (0.82-0.89)
Race						
Black	14,612 (13,591-15,633)	9,862	0.67 (0.63-0.73)	17,259 (15,834-18,684)	13,845	0.80 (0.74-0.87)
Hispanic	5,989 (5,243-6,735)	4,402	0.74 (0.65-0.84)	6,949 (5,895-8,003)	6,414	0.92 (0.80-1.09)
White	29,030 (28,262-29,798)	21,147	0.73 (0.71-0.75)	34,571 (33,490-35,651)	27,922	0.81 (0.78-0.83)
Other[a]	8,556 (7,520-9,592)	5,531	0.65 (0.58-0.74)	10,182 (8,719-11,645)	8,375	0.82 (0.72-0.96)
Sex						
Male	22,441 (21,632-23,250)	16,158	0.72 (0.69-0.75)	26,608 (25,528-27,688)	22,284	0.84 (0.80-0.87)
Female	20,258 (19,611-20,905)	14,420	0.71 (0.69-0.74)	24,083 (23,176-24,990)	18,805	0.78 (0.75-0.81)
PCP						
No	20,157 (19,561-20,753)	14,392	0.71 (0.69-0.74)	24,011 (23,172-24,850)	19,775	0.82 (0.82-0.85)
Yes	23,473 (22,530-22,416)	17,160	0.73 (0.70-0.76)	27,802 (26,481-29,123)	21,965	0.79 (0.75-0.83)
Comorbidity score						
<0	25,388 (24,475-26,301)	18,095	0.71 (0.69-0.74)	30,193 (28,909-31,477)	24,384	0.81 (0.77-0.84)
0	14,600 (13,822-15,378)	9,077	0.62 (0.59-0.66)	17,376 (16,284-18,468)	14,199	0.82 (0.77-0.87)
>0	22,143 (21,253-23,033)	17,104	0.77 (0.74-0.71)	26,154 (24,902-27,406)	22,314	0.85 (0.81-0.90)

[a]Other includes Asian/Pacific Islander, American Indian/Alaska native, or others.
CI: confidence interval; O/E ratio: observed/expected ratio; PCP: primary care provider.

© 2023 Doan C et al. *JAMA Network Open.*

Supplementary eTable 13. Stratified analysis for non-Medicaid beneficiaries. Expected and observed low dose computed tomography (LDCT) rates for 3/20 to 2/21 and 3/21 to 2/22 from a prediction model using 2017-2019 cohorts, with a linear probability model for each sub-cohort, with year as the only variable in the models.

Non-Medicaid	Expected LDCT rate per 1,000,000 (95% CI)	Observed LDCT rate per 1,000,000	O/E ratio (95% CI)	Expected LDCT rate per 1,000,000 (95% CI)	Observed LDCT rate	O/E ratio (95% CI)
All	14,008 (13,845-14,171)	10,886	0.78 (0.77-0.79)	16,567 (16,336-16,798)	14,546	0.88 (0.86-0.89)
Age, years						
50-65	22,769 (22,040-23,498)	17,326	0.76 (0.74-0.79)	27,042 (26,018-28,066)	23,063	0.85 (0.82-0.89)
66-74	13,274 (13,109-13,439)	10,386	0.78 (0.77-0.79)	15,711 (15,472-15,950)	13,956	0.89 (0.88-0.90)
Race						
Black	9,137 (8,612-9,662)	7,259	0.79 (0.75-0.84)	10,762 (10,020-11,504)	10,398	0.97 (0.90-1.04)
Hispanic	5,838 (5,286-6,391)	4,044	0.69 (0.63-0.77)	6,893 (6,113-7,673)	6,376	0.92 (0.83-1.04)
White	15,044 (14,851-15,237)	11,677	0.78 (0.77-0.79)	17,790 (17,533-18,047)	15,479	0.87 (0.86-0.88)
Other[a]	9,628 (9,055-10,202)	7,583	0.79 (0.74-0.84)	11,439 (10,632-12,247)	10,400	0.91 (0.85-0.98)
Sex						
Male	15,752 (15,499-16,005)	12,357	0.78 (0.77-0.80)	18,639 (18,282-18,996)	16,271	0.87 (0.86-0.89)
Female	12,509 (12,297-12,721)	9,625	0.78 (0.77-0.80)	14,793 (14,495-15,091)	13,085	0.88 (0.87-0.90)
PCP						
No	12,878 (12,690-13,066)	9,961	0.77 (0.76-0.78)	15,293 (15,028-15,558)	13,791	0.90 (0.89-0.92)
Yes	16,677 (16,354-17,000)	13,127	0.79 (0.77-0.80)	19,663 (19,208-20,118)	17,082	0.87 (0.85-0.90)
Comorbidity score						
<0	15,570 (15,243-15,897)	12,241	0.79 (0.77-0.80)	18,409 (17,944-18,874)	16,616	0.90 (0.88-0.93)
0	9,592 (9,392-9,792)	7,071	0.74 (0.72-0.75)	11,289 (11,009-11,569)	10,260	0.91 (0.89-0.93)
>0	20,333 (19,943-20,723)	16,215	0.80 (0.78-0.81)	24,050 (23,499-24,601)	21,046	0.88 (0.86-0.90)

[at]Other includes Asian/Pacific Islander, American Indian/Alaska native, or others.
CI: confidence interval; O/E ratio: observed/expected ratio; PCP: primary care provider.

© 2023 Doan C et al. *JAMA Network Open.*
