# Supplementary material for: Breast and Lung Cancer Screening Among Medicare Enrollees During the COVID-19 Pandemic
Source: JAMA Netw Open. 2023 Feb 3;6(2):e2255589. doi: 10.1001/jamanetworkopen.2022.55589 (PMC9898823; doi:10.1001/jamanetworkopen.2022.55589)
Supplement: Supplement 2. — Data Sharing Statement [file jamanetwopen-e2255589-s002.pdf]

Data Sharing Statement

Doan. Breast and Lung Cancer Screening Among Medicare Enrollees During the COVID-19 Pandemic. *JAMA Netw Open*. Published February 03, 2023.
doi:10.1001/jamanetworkopen.2022.55589

Data
Data available: No

Additional Information
Explanation for why data not available: This data is covered by a Data Use Agreement, which researchers will need to complete to access the data.
